# Second-line tislelizumab versus chemotherapy in Japanese patients with advanced or metastatic esophageal squamous cell carcinoma: subgroup analysis from RATIONALE-302

**DOI:** 10.1007/s10388-023-01040-w

**Published:** 2024-01-19

**Authors:** Hiroki Hara, Taroh Satoh, Takashi Kojima, Takahiro Tsushima, Yu Sunakawa, Morihito Okada, Ningning Ding, Hongqian Wu, Liyun Li, Tian Yu, Gisoo Barnes, Ken Kato

**Affiliations:** 1 Department of Gastroenterology, Saitama Cancer Center, Saitama, Japan; 2 Osaka University Hospital, Suita, Japan; 3 National Cancer Center Hospital East, Chiba, Japan; 4 Division of Gastrointestinal Oncology, Shizuoka Cancer Center, Shizuoka, Japan; 5 Department of Clinical Oncology, St. Marianna University School of Medicine, Kanagawa, Japan; 6 Department of Surgical Oncology, Hiroshima University, Hiroshima, Japan; 7 BeiGene, Ltd, Zhongguancun Life Science Park, Beijing, China; 8 BeiGene, Ltd, Ridgefield Park, NJ USA; 9 Clinical Pharmacology, BeiGene USA, Inc., San Mateo, CA USA; 10 Department of Head and Neck Esophageal Medical Oncology, National Cancer Center Hospital, Tokyo, Japan

**Keywords:** Esophageal squamous cell carcinoma, Tislelizumab, Japan, Programmed cell death protein 1 inhibitor

## Abstract

**Background:**

Esophageal squamous cell carcinoma (ESCC) has a poor prognosis, with limited second-line systemic therapy options, and represents an increasing disease burden in Japan. In the phase 3 RATIONALE-302 study, the anti-programmed cell death protein 1 antibody, tislelizumab, significantly improved overall survival (OS) versus chemotherapy as second-line treatment for advanced/metastatic ESCC. Here, we report the Japanese patient subgroup results.

**Methods:**

Patients with advanced/metastatic ESCC, with disease progression during/after first-line systemic therapy were randomized 1:1 to open-label tislelizumab 200 mg every 3 weeks or investigator’s choice of chemotherapy (paclitaxel/docetaxel). Efficacy and safety were assessed in all randomized Japanese patients.

**Results:**

The Japanese subgroup comprised 50 patients (*n* = 25 per arm). Tislelizumab improved OS versus chemotherapy (median: 9.8 vs. 7.6 months; HR 0.59; 95% CI 0.31, 1.12). Among patients with programmed death-ligand 1 score ≥ 10%, median OS was 12.5 months with tislelizumab (*n* = 10) versus 2.9 months with chemotherapy (*n* = 6) (HR 0.31; 95% CI 0.09, 1.03). Tislelizumab improved progression-free survival versus chemotherapy (median: 3.6 vs. 1.7 months, respectively; HR 0.50; 95% CI 0.27, 0.95). Objective response rate was greater with tislelizumab (32.0%) versus chemotherapy (20.0%), and responses were more durable (median duration of response: 8.8 vs. 2.6 months, respectively). Fewer patients experienced ≥ grade 3 treatment-related adverse events with tislelizumab (24.0%) versus chemotherapy (47.8%). Tislelizumab demonstrated an improvement in health-related quality of life versus chemotherapy.

**Conclusions:**

As second-line therapy for advanced/metastatic ESCC, tislelizumab improved OS versus chemotherapy, with a favorable safety profile, in the Japanese patient subgroup, consistent with the overall population.

**Clinical trial registry:**

ClinicalTrials.gov: NCT03430843.

**Supplementary Information:**

The online version contains supplementary material available at 10.1007/s10388-023-01040-w.

## Introduction

In 2020, esophageal cancer (EC) was ranked the eighth most commonly diagnosed cancer and sixth most common cause of cancer death worldwide [1]. The burden of EC is greatest in Eastern Asia, with the highest global incidences reported in this region for both men and women [2, 3]. In Japan, there were over 26,000 newly diagnosed cases of EC in 2020, along with over 12,000 deaths caused by EC [4]. Esophageal squamous cell carcinoma (ESCC) is the predominant histological subtype in Japan, which accounts for over 90% of EC cases [5].

Second-line treatment strategies after initial chemotherapy for advanced ESCC have historically been limited, representing a significant unmet need in this patient population [6]. Notably, a real-world study of treatment patterns among a sample of patients with ESCC from Japan in 2018 found that only 54% of patients with advanced disease received active second-line treatment (typically taxane-based chemotherapy), with the remainder receiving best supportive care [6]. More recently, trials of drugs targeting the programmed cell death protein 1 (PD-1)/programmed death-ligand 1 (PD-L1) pathway have demonstrated prolonged survival with anti-PD-1 antibodies versus chemotherapy in patients with advanced or metastatic ESCC whose disease progressed after first-line systemic therapy [7–9]. Subgroup analyses of these studies have shown that this survival benefit is also applicable to Japanese patients treated with anti-PD-1 antibodies [10, 11]. In these studies, second-line therapy with either nivolumab or pembrolizumab demonstrated favorable efficacy compared with chemotherapy (median overall survival 13.4 vs. 9.4 months with nivolumab vs. chemotherapy, HR 0.77; 12.4 vs. 8.2 months with pembrolizumab vs. chemotherapy, HR 0.68) [10, 11]. Tislelizumab is a humanized IgG4 anti-PD-1 monoclonal antibody with high affinity for PD-1 and has demonstrated antitumor activity in patients with ESCC and gastroesophageal junction adenocarcinoma, either alone or in combination with chemotherapy in clinical trials [12–15]. In the international, randomized, phase 3 RATIONALE-302 study (NCT03430843) that enrolled patients with advanced or metastatic ESCC, tislelizumab monotherapy as second-line treatment demonstrated a statistically significant improvement in overall survival (OS) compared with chemotherapy (median OS: 8.6 vs. 6.3 months, respectively; hazard ratio [HR] 0.70, *P* = 0.0001) [14]. Here, we report the results of a subgroup analysis conducted in Japanese patients from the RATIONALE-302 study.

## Methods

### Trial design, treatment, and participants

Full details of the study design and methodology for RATIONALE-302 have been previously described [14]. Briefly, this was an open-label, randomized, active-controlled, global, phase 3 clinical study to compare the efficacy and safety of tislelizumab versus chemotherapy as second-line treatment in patients with advanced or metastatic ESCC, recruiting patients across 11 countries, including Japan. Patients were stratified by region of enrollment (Asia excluding Japan vs. Japan vs. United States of America/Europe), an Eastern Cooperative Oncology Group (ECOG) performance status of 0 or 1, and investigator’s choice of single-agent chemotherapy (docetaxel, paclitaxel, or irinotecan). Eligible patients had histologically confirmed, locally advanced or metastatic ESCC whose disease progressed during or after first-line systemic therapy, or during or within 6 months after definitive chemoradiotherapy, neo-adjuvant or adjuvant therapy. The study excluded patients who had received ≥ 2 lines of systemic therapy for advanced or metastatic disease or had previously received treatment with a PD-1 or PD-L1 inhibitor. Full inclusion and exclusion criteria are described in the Supplementary Materials.

Eligible patients in the overall study population were randomized (1:1) to receive tislelizumab or investigator's choice of single-agent chemotherapy, which included docetaxel, paclitaxel, or irinotecan. However, as irinotecan is not approved in Japan, investigator's choice of single-agent chemotherapy was restricted to either docetaxel or paclitaxel for the Japanese subgroup. Tislelizumab 200 mg was administered intravenously (IV) every 3 weeks (Q3W). In the chemotherapy arm, paclitaxel 100 mg/m^2^ was administered IV once weekly for 6 weeks, followed by 1 week rest; docetaxel 70 mg/m^2^ was administered IV Q3W. Cross-over between chemotherapy regimens or between the chemotherapy and tislelizumab treatment arms was not allowed during the study treatment period.

### Endpoints and assessments

The primary endpoint was OS in the intent-to-treat (ITT) population, which included all randomized patients. The key secondary endpoint was OS in the PD-L1-positive population, with PD-L1 positivity defined as a PD-L1 score ≥ 10%; details of how PD-L1 score was determined have been previously published [14]. Other secondary efficacy endpoints included progression-free survival (PFS), objective response rate (ORR), and duration of response (DoR), all assessed by investigators per Response Evaluation Criteria in Solid Tumors (RECIST) version 1.1, and patient-reported health-related quality of life (HRQoL) outcomes, as previously described for the overall study population [14].

Safety was assessed as a secondary endpoint through monitoring of the incidence and severity of treatment-emergent adverse events (TEAEs) according to National Cancer Institute Common Terminology Criteria for Adverse Events version 4.03. Safety analyses were performed in the safety population, which included all patients who received at least one dose of any study drug.

Further detail on the study endpoints and their assessment is provided in the Supplementary Materials.

### Statistical analyses

Sample size calculations and statistical considerations for the primary analyses in the overall study population have been reported previously [14]. No formal hypothesis testing was performed for the Japanese patient subgroup analysis and all statistical analyses reported herein are descriptive.

Median OS was estimated by the Kaplan–Meier method with two-sided 95% confidence intervals (CIs) estimated using the method of Brookmeyer and Crowley. HRs for OS analyses were estimated using an unstratified Cox regression model, including only treatment as a covariate, accompanied by two-sided 95% CIs. PFS and DoR were analyzed using similar methodology to OS. ORR was calculated, accompanied by two-sided 95% CIs calculated using the Clopper-Pearson method. In addition, the odds ratio for ORR was calculated, accompanied by two-sided 95% CIs. Changes from baseline to Cycle 4 in patient-reported HRQoL assessments and safety outcomes were analyzed using descriptive statistics.

## Results

### Patients and treatment

Of the 512 patients randomized in the RATIONALE-302 study, 50 were enrolled in Japan and evenly randomized to tislelizumab (*n* = 25) or chemotherapy (*n* = 25), constituting the Japanese patient subgroup (Fig. S1).

Within the Japanese patient subgroup, all (25/25) patients in the tislelizumab arm and 23/25 (92.0%) patients in the chemotherapy arm (docetaxel: *n* = 6; paclitaxel: *n* = 17) received study treatment; in the chemotherapy arm, 2 patients did not receive treatment due to withdrawal. The median age was 65.0 years, most patients were male (39/50; 78.0%) and former/current smokers (45/50; 90.0%). Patient demographics and baseline characteristics were generally comparable between treatment arms, with the exception that more patients in the tislelizumab arm compared with the chemotherapy arm were ≥ 65 years of age (68.0% [17/25] and 36.0% [9/25], respectively), and had PD-L1 score ≥ 10% (40.0% [10/25] and 24.0% [6/25], respectively), prior surgery (44.0% [11/25] vs. 32.0% [8/25], respectively), and prior radiotherapy (80.0% [20/25] vs. 60.0% [15/25], respectively) (Table 1). In addition, in the tislelizumab arm, all patients (25/25) had metastatic disease at study entry, whereas in the chemotherapy arm, 16.0% (4/25) had locally advanced disease, and the remainder had metastatic disease. Patient demographics and baseline characteristics for the overall population are shown in Table S1.

**Table 1:** Patient demographics and baseline characteristics in the Japanese patient subgroup

	Tislelizumab (*n* = 25)	Chemotherapy (*n* = 25)
Age		
Median, years (range)	67.0 (47–83)	63.0 (52–77)
< 65 years, *n* (%)	8 (32.0)	16 (64.0)
≥ 65 years, *n* (%)	17 (68.0)	9 (36.0)
Sex, *n* (%)		
Male	20 (80.0)	19 (76.0)
Female	5 (20.0)	6 (24.0)
ECOG performance status, *n* (%)		
0	14 (56.0)	14 (56.0)
1	11 (44.0)	11 (44.0)
PD-L1 score^a^, *n* (%)		
≥ 10%	10 (40.0)	6 (24.0)
< 10%	4 (16.0)	6 (24.0)
Missing	11 (44.0)	13 (52.0)
Smoking status, *n* (%)		
Never	3 (12.0)	2 (8.0)
Former/current	22 (88.0)	23 (92.0)
Previous anti-cancer interventions/therapies,* n* (%)		
Surgery	11 (44.0)	8 (32.0)
Radiotherapy	20 (80.0)	15 (60.0)
Platinum-based chemotherapy	23 (92.0)	25 (100.0)
Disease stage at study entry, *n* (%)		
Locally advanced	0 (0.0)	4 (16.0)
Metastatic	25 (100.0)	21 (84.0)

Data are presented for the Japanese patient subgroup of the intent-to-treat population, which comprised all randomized patients, analyzed according to their randomized treatment arm

*ECOG* Eastern Cooperative Oncology Group, *PD-L1* programmed death-ligand 1

^a^Visually estimated combined positive score; ‘missing’ score referred to patients without sample collection, not evaluable at baseline or scored with unqualified sample

As of final analysis data cutoff on December 1, 2020, the median study follow-up time in the Japanese patient subgroup was 9.8 months (range 2.7–22.0) for tislelizumab and 6.1 months (range 0.2–20.3) for chemotherapy. Treatment had been discontinued in 88.0% (22/25) of patients in the tislelizumab arm and in all (23/23) treated patients in the chemotherapy arm (Fig. S1). The most common primary reason for study drug discontinuation was disease progression in both treatment arms. Median duration of exposure was longer for tislelizumab (126.0 days [range 21–670]) than for chemotherapy (66.5 days [range 42–108] for docetaxel; 49.0 days [range 21–357] for paclitaxel).

In the Japanese patient subgroup, post-study treatment anti-cancer therapies were received by 68.0% (17/25) of patients in the tislelizumab arm and 48.0% (12/25) in the chemotherapy arm (Table S2). Subsequent anti-cancer therapy included immunotherapy for 16.0% (4/25) and 24.0% (6/25) of patients in the tislelizumab and chemotherapy arms, respectively.

### Efficacy

#### Overall survival

**Fig. 1:**
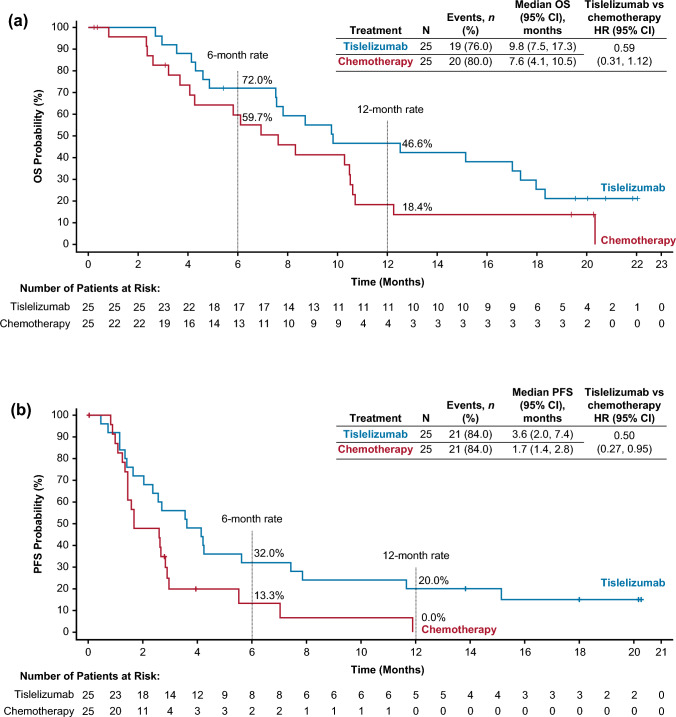
Overall survival (**a**) and progression-free survival (**b**) in the Japanese patient subgroup. *CI* confidence interval, *HR* hazard ratio, *OS* overall survival, *PFS* progression-free survival. Data are presented for the Japanese patient subgroup of the intent-to-treat population, which comprised all randomized patients, analyzed according to their randomized treatment arm. The graph presents Kaplan–Meier survival plots, with OS rates (cumulative probability of OS) and PFS rates (cumulative probability of PFS) at selected timepoints estimated by Kaplan–Meier method. The tabular data present hazard ratios based on an unstratified Cox regression model including treatment as a covariate; medians estimated by the Kaplan–Meier method, with 95% CIs estimated using the Brookmeyer and Crowley method

Tislelizumab improved OS compared with chemotherapy in the Japanese patient subgroup (Fig. 1a). The OS HR for tislelizumab versus chemotherapy was 0.59 (95% CI 0.31, 1.12), and median OS was 9.8 months (95% CI 7.5, 17.3) vs. 7.6 months (95% CI 4.1, 10.5). Among the patients with PD-L1 score ≥ 10%, the OS HR for tislelizumab versus chemotherapy was 0.31 (95% CI 0.09, 1.03), with median OS of 12.5 months (95% CI 4.3, upper limit not evaluable [NE]) and 2.9 months (95% CI 2.3, NE), respectively (Fig. S2 and Table S3). OS results in the overall patient population are shown in Table S3.

#### Progression-free survival

Tislelizumab improved PFS compared with chemotherapy in the Japanese patient subgroup (Fig. 1b). The PFS HR for tislelizumab versus chemotherapy was 0.50 (95% CI 0.27, 0.95), and median PFS was 3.6 months (95% CI 2.0, 7.4) vs. 1.7 months (95% CI 1.4, 2.8). PFS results in the overall patient population are shown in Table S3.

#### Tumor response

Tislelizumab was associated with higher ORR in the Japanese patient subgroup compared with chemotherapy (32.0% [95% CI 14.9, 53.5] vs. 20.0% [95% CI 6.8, 40.7]) (Table S3). There was one complete response in both arms, more partial responses in the tislelizumab arm than in the chemotherapy arm, and a higher rate of stable disease in the tislelizumab arm than in the chemotherapy arm (Table S3). Responses were more durable with tislelizumab versus chemotherapy, with a median DoR of 8.8 months (95% CI 2.9, NE) vs. 2.6 months (95% CI 1.1, NE) (Table S3). Tumor response results for the overall patient population are shown in Table S3.

#### Health-related quality of life

Based on changes from baseline to Cycle 4, HRQoL scores in the Japanese patient subgroup tended to be maintained or improved with tislelizumab versus chemotherapy (Fig. S3). Changes in European Organisation for Research and Treatment of Cancer (EORTC) Core Quality of Life Questionnaire (QLQ-C30), key PRO endpoint scale scores indicated maintenance (i.e., < 5-point change from baseline) in overall global health status/quality of life (GHS/QoL), physical functioning and fatigue scores with tislelizumab, compared with worsening in these scores with chemotherapy. Similarly, changes in the key PRO endpoints (main ESCC symptoms) measured by the EORTC Quality of Life Questionnaire esophageal cancer module (QLQ-EOS18) scores indicated either maintenance or less worsening in dysphagia, eating, reflux, and pain with tislelizumab versus chemotherapy. Changes in EuroQol 5D (EQ-5D-5L) visual analogue scale scores indicated an improvement with tislelizumab that was not clinically meaningful when compared with worsening scores with chemotherapy. Results for the overall patient population are shown in Fig. S3.

### Safety and tolerability

In the Japanese patient subgroup, most patients in both the tislelizumab and chemotherapy treatment arms experienced at least one TEAE (96.0% [24/25] and 95.7% [22/23], respectively) (Table 2). The proportion of patients experiencing treatment-related TEAEs (TRAEs) in the Japanese patient subgroup was noticeably lower in the tislelizumab arm (68.0%; 17/25) compared with the chemotherapy arm (95.7%; 22/23) (Table 2). Fewer patients experienced ≥ grade 3 TRAEs in the tislelizumab arm (24.0% [6/25]) versus the chemotherapy arm (47.8% [11/23]). Serious TRAEs were reported in 16.0% (4/25) vs. 8.7% (2/23) of patients in the tislelizumab versus chemotherapy arms, respectively. The proportions of patients experiencing TRAEs leading to treatment discontinuation were similar in the tislelizumab and chemotherapy arms (8.0% [2/25] and 8.7% [2/23], respectively). Fewer patients required dose modification due to TRAEs in the tislelizumab arm (16.0%; 4/25) than in the chemotherapy arm (69.6%; 16/23) (Table S4). The most commonly reported TRAEs in the tislelizumab arm were fatigue (in 12.0% [3/25] of patients vs. 21.7% [5/23] with chemotherapy), hypothyroidism (12.0% [3/25] vs. 0%), malaise (12.0% [3/25] vs. 17.4% [4/23]), pneumonitis (12.0% [3/25] vs. 8.7% [2/23]), and pruritus (12.0% [3/25] vs. 4.3% [1/23]). In the chemotherapy arm, the most common TRAEs were neutrophil count decreased (in 56.5% [13/23] of patients versus 0% with tislelizumab), white blood cell count decreased (52.2% [12/23] vs. 4.0% [1/25]), alopecia (47.8% [11/23] vs. 0% [0/25]), and peripheral sensory neuropathy (30.4% [7/23] vs. 0%; Table 2). Three patients (12.0%) experienced an infusion-related reaction in the tislelizumab arm (Table S5).

**Table 2:** Summary of overall adverse event incidence in the Japanese patient subgroup

*n* (%)	Tislelizumab (*n* = 25)	Chemotherapy (*n* = 23)
Overall TEAE incidence		
Any TEAE	24 (96.0)	22 (95.7)
≥ Grade 3 TEAE	11 (44.0)	16 (69.6)
Serious TEAE	9 (36.0)	10 (43.5)
TEAE leading to treatment discontinuation	2 (8.0)	4 (17.4)
TEAE leading to dose modification^a^	8 (32.0)	16 (69.6)
TEAE leading to death^b^	0 (0.0)	0 (0.0)
Any TRAE	17 (68.0)	22 (95.7)
≥ Grade 3 TRAE	6 (24.0)	11 (47.8)
Serious TRAE	4 (16.0)	2 (8.7)
TRAE leading to treatment discontinuation	2 (8.0)	2 (8.7)
TRAE leading to dose modification^a^	4 (16.0)	16 (69.6)
TRAE leading to death^b^	0 (0.0)	0 (0.0)
Incidence of most common TRAEs occurring in ≥ 10% of patients in either treatment arm by preferred term^c^		
Fatigue	3 (12.0)	5 (21.7)
Hypothyroidism	3 (12.0)	0 (0.0)
Malaise	3 (12.0)	4 (17.4)
Pneumonitis	3 (12.0)	2 (8.7)
Pruritus	3 (12.0)	1 (4.3)
Arthralgia	2 (8.0)	6 (26.1)
Stomatitis	1 (4.0)	6 (26.1)
White blood cell count decreased	1 (4.0)	12 (52.2)
Alopecia	0 (0.0)	11 (47.8)
Decreased appetite	0 (0.0)	5 (21.7)
Myalgia	0 (0.0)	3 (13.0)
Neutrophil count decreased	0 (0.0)	13 (56.5)
Peripheral sensory neuropathy	0 (0.0)	7 (30.4)

All data are presented as number of patients with at least one event (percentage of patients). Data are presented for the Japanese subgroup of the safety population, which comprised all randomized patients who received at least one dose of a study drug, analyzed according to the actual study drug received. TRAEs include TEAEs that were considered by the investigator to be related to study drug or TEAEs with a missing causality. Adverse event grades were based on National Cancer Institute Common Terminology Criteria for Adverse Events version 4.03. Adverse events were coded using Medical Dictionary for Regulatory Activities version 23.0

*TEAE* treatment-emergent adverse event, *TRAE* treatment-related treatment-emergent adverse event

^a^Dose modification included dose held, dose interruption and dose reduction for the chemotherapy arm, and dose held and dose interruption for the tislelizumab arm

^b^Deaths caused by disease progression were excluded

^c^Ordered by decreasing incidence in the tislelizumab arm

The incidence of TEAEs and TRAEs in the overall patient population is presented in Table S6.

### Pharmacokinetics

Tislelizumab serum concentrations were similar for the Japanese patient subgroup and the overall patient population, with overlapping concentration ranges (Table S7).

## Discussion

Consistent with results from the overall population of RATIONALE-302 [16], tislelizumab improved OS compared with chemotherapy as second-line treatment in Japanese patients with advanced or metastatic ESCC. In the Japanese patient subgroup, a favorable improvement in PFS was also seen with tislelizumab compared with chemotherapy, together with greater and more durable antitumor responses, and improvements in HRQoL scores, as seen in the overall study patient population [14].

Tislelizumab pharmacokinetic profiles in the Japanese patient subgroup were generally consistent with findings in the overall RATIONALE-302 patient population. This corroborates findings from a population PK modeling study, which concluded that race is not a significant covariate of tislelizumab pharmacokinetics [17]; thus, there is no clinically relevant impact of Japanese ethnicity on tislelizumab pharmacokinetic profiles.

The baseline characteristics of the Japanese patient subgroup were generally similar to those of the overall population of RATIONALE-302, except that more patients in the Japanese subpopulation had an ECOG performance status of 0 compared with the overall population in both treatment arms (tislelizumab arm: Japan subgroup 56.0% versus overall population 25.8%; chemotherapy arm: Japan subgroup 56.0% versus overall population 23.4%) [14]. This observation of better functional performance status in the Japanese subpopulation compared with the global overall population of RATIONALE-302 is consistent with findings in other studies investigating anti-PD-1 antibodies as monotherapy in second-line treatment of advanced esophageal tumors, or in combination with chemotherapy or other immunotherapies in first-line treatment [10, 11, 18]. In the Japanese subgroup of RATIONALE-302, median OS in both the tislelizumab and chemotherapy arms was numerically longer than in the overall population [14]. The observed differences in baseline ECOG performance status between the Japanese subgroup and the overall population may have in part contributed to the numerically better OS outcomes in the Japanese subpopulation. Compared to other randomized trials that assessed the use of immune checkpoint inhibitors as second-line therapy for advanced/metastatic ESCC, treatment with tislelizumab demonstrated an OS improvement over chemotherapy (median OS 9.8 vs. 7.6 months, HR 0.59) that was broadly similar to nivolumab versus chemotherapy (median OS 13.4 vs. 9.4 months, HR 0.77) and pembrolizumab versus chemotherapy (12.4 vs. 8.2 months, HR 0.68) in the Japanese patients [10, 11], though caution should be taken when comparing subgroup results across studies.

In the Japanese patient subgroup of RATIONALE-302, the improvements in OS and PFS with tislelizumab versus chemotherapy appeared numerically greater among patients with baseline PD-L1 score ≥ 10% compared with the total Japanese patient subgroup, in line with observations from a similar study of pembrolizumab [11]. However, findings based on baseline PD-L1 expression status should be interpreted cautiously because of the very limited sample size of patients with PD-L1 score ≥ 10%, and the imbalance in PD-L1 expression status between treatment arms.

Previous subanalyses of other phase 3 trials have also reported prolonged survival with PD-1 inhibitor monotherapy versus chemotherapy as second-line treatment for advanced ESCC in Japanese patients [10, 11]. Based on the results of these trials, both nivolumab and pembrolizumab are approved in Japan for the treatment of unresectable, advanced or recurrent ESCC that has progressed following chemotherapy [19], although pembrolizumab use is restricted to patients with PD-L1-positive tumors [17, 20]. Following these approvals, PD-1 inhibitor monotherapy has become the standard second-line treatment for advanced or metastatic ESCC [20, 21]. The baseline characteristics of the Japanese patient subgroup in RATIONALE-302 were comparable with those of the Japanese patient subgroups in the previously reported PD-1 inhibitor trials in this setting [10, 11], supporting the relevance and applicability of our findings to Japanese patients with advanced or metastatic ESCC.

The safety profile of tislelizumab was more favorable compared with that of chemotherapy, with no unexpected safety signals identified in the Japanese patient subgroup, similar to observations from analyses of treatment of Japanese patients with nivolumab and pembrolizumab [10, 11]. Consistent with the results in the overall study patient population [14], fewer patients in the Japanese patient subgroup experienced any TRAEs or ≥ grade 3 TRAEs with tislelizumab compared with chemotherapy. By preferred term, no TRAEs were reported in more than 3 of the 25 patients in the Japanese patient subgroup in the tislelizumab arm, and the spectrum of TEAEs reported was broadly consistent with that seen in the overall study patient population [14]. These favorable findings with tislelizumab monotherapy are supported by the HRQoL outcomes indicated via ESCC-specific patient-reported symptoms.

It must be noted that the RATIONALE-302 study and this subgroup analysis are subject to various limitations, including an open-label study design and lack of blinded independent review of tumor responses, which may have impacted response data (ORR, PFS, and DoR). Limitations of the present analysis in this Japanese patient subgroup include the small sample size, and the fact that these exploratory analyses are descriptive only.

In conclusion, among Japanese patients with advanced or metastatic ESCC whose disease progressed during or after first-line systemic therapy, tislelizumab prolonged OS versus chemotherapy, with a manageable safety profile. These results were consistent with the results seen in the overall study population and warrant consideration of tislelizumab as a second-line treatment option for patients with advanced or metastatic ESCC in Japan.

### Supplementary Information

Below is the link to the electronic supplementary material.

Supplementary file1 (DOCX 341 KB)

## Data Availability

On request, and subject to certain criteria, conditions, and exceptions, BeiGene, Ltd, will provide access to individual deidentified participant data from BeiGene-sponsored global interventional clinical studies conducted for medicines (1) for indications that have been approved or (2) in programs that have been terminated. BeiGene will also consider requests for the protocol, data dictionary, and statistical analysis plan. Data requests may be submitted to DataDisclosure@beigene.com.
