# Supplementary material for: Second-line tislelizumab versus chemotherapy in Japanese patients with advanced or metastatic esophageal squamous cell carcinoma: subgroup analysis from RATIONALE-302
Source: Esophagus. 2024 Jan 19;21(2):102–10. doi: 10.1007/s10388-023-01040-w (PMC10957685; doi:10.1007/s10388-023-01040-w)
Supplement: Supplementary file 1 — Supplementary file1 (DOCX 341 KB) [file 10388_2023_1040_MOESM1_ESM.docx]

**Title:** Second-line tislelizumab versus chemotherapy in Japanese patients with advanced or metastatic esophageal squamous cell carcinoma: subgroup analysis from RATIONALE-302

**Authors:**

Hiroki Hara,^1^ Taroh Satoh,^2^ Takashi Kojima,^3^ Takahiro Tsushima,^4^ Yu Sunakawa,^5^ Morihito Okada,^6^ Ningning Ding,^7^ Hongqian Wu,^8^ Liyun Li,^7^ Tian Yu,^9^ Gisoo Barnes,^8^ Ken Kato^10^

**Affiliations:**

^1^Department of Gastroenterology, Saitama Cancer Center, Saitama, Japan;

^2^Osaka University Hospital, Suita, Japan;

^3^National Cancer Center Hospital East, Chiba, Japan;

^4^Division of Gastrointestinal Oncology, Shizuoka Cancer Center, Shizuoka, Japan;

^5^Department of Clinical Oncology, St. Marianna University School of Medicine, Kanagawa, Japan;

^6^Hiroshima University, Hiroshima, Japan;

^7^BeiGene, Ltd, Zhongguancun Life Science Park, Beijing, China;

^8^BeiGene, Ltd, Ridgefield Park, NJ, USA;

^9^Clinical Pharmacology, BeiGene USA, Inc., San Mateo, CA, USA;

^10^Department of Head and Neck Esophageal Medical Oncology, National Cancer Center Hospital, Tokyo, Japan

**Corresponding author:** Ken Kato

Address: Department of Head and Neck Esophageal Medical Oncology, National Cancer Center Hospital, Tokyo, Japan

Email: kenkato@ncc.go.jp

**Target journal:** *Esophagus*

**Supplementary Material**

**Contents**

| Investigators list for Japan patient subgroup |
| --- |
| Full inclusion and exclusion criteria |
| Study endpoints |
| Supplementary tables |
| **Table S1** Patient demographics and baseline characteristics in the Japanese subgroup and overall study patient population |
| **Table S2** Post-study treatment anti-cancer interventions/therapies in the Japanese patient subgroup |
| **Table S3** Summary of efficacy outcomes in the overall study patient population and Japanese patient subgroup |
| **Table S4** Treatment-related treatment-emergent adverse events leading to dose modification in the Japanese patient subgroup |
| **Table S5** Infusion-related reactions in the Japanese patient subgroup |
| **Table S6** Summary of treatment-emergent adverse event incidence in the overall study patient population and Japanese patient subgroup |
| **Table S7** Tislelizumab serum concentration in the tislelizumab arm in the overall study patient population and Japanese patient subgroup |
| Supplementary figures |
| **Fig. S1** Patient flow in the Japanese patient subgroup |
| **Fig. S2** Overall survival in the PD-L1-positive population for the Japanese patient subgroup |
| **Fig. S3** Observed scores and change from baseline in QLQ-C30 scores (**a**), QLQ-OES18 scores (**b**) and EQ-5D-5L scores (**c**) at Cycle 4 (or Week 12) in the overall study patient population and Japanese patient subgroup |

## Investigators list for Japan patient subgroup

| **Principal investigators** |
| --- |
| Keisho Chin |
| Masahiro Goto |
| Hiroki Hara |
| Ryu Ishihara |
| Shigenori Kadowaki |
| Ken Kato (SC member) |
| Takashi Kojima |
| Keiko Minashi |
| Masaru Morita |
| Satoru Motoyama |
| Manabu Muto |
| Tomohiro Nishina |
| Morihito Okada |
| Taroh Satoh |
| Yuichi Shibuya |
| Yu Sunakawa |
| Takao Tamura |
| Masahiro Tsuda |
| Akihito Tsuji |
| Takahiro Tsushima |
| Hisateru Yasui |

**Full inclusion and exclusion criteria**

Inclusion criteria

To be eligible to participate in this study, a patient was required to meet all of the following criteria:

1. Is male or female, aged ≥ 18 years on the day the patient voluntarily agrees to participate in the study (or acceptable age according to local regulations, whichever is older)
2. Able to provide written informed consent and can understand and agree to comply with the requirements of the study and the schedule of assessments
3. Histologically confirmed diagnosis of esophageal squamous cell carcinoma (ESCC)
4. Tumor progression during or after first-line systemic treatment for advanced unresectable/metastatic ESCC

NOTE: Patients with disease progression that occurs during treatment or within 6 months (180 days) of cessation of neoadjuvant/adjuvant treatment (chemotherapy or chemoradiotherapy) are eligible provided all other criteria are met

NOTE: A line of treatment begins with the administration of the first agent in a regimen and ends with disease progression. A line of therapy is preserved when chemotherapy is switched due to toxicities

1. At least one measurable/evaluable lesion by Response Evaluation Criteria in Solid Tumors (RECIST) version 1.1 as determined by the local site investigator/radiology assessment within 28 days prior to randomization

NOTE: Lesions that have been previously irradiated may be considered evaluable provided there is evidence of disease progression following the completion of radiation therapy

1. Eastern Cooperative Oncology Group (ECOG) performance status of 0 or 1 prior to randomization
2. Laboratory data meeting the criteria below within 14 days prior to randomization. Laboratory data will not be valid if the patient has received growth factors or blood transfusion for prophylactic use within 7 days before the laboratory testing:
   - - Absolute neutrophil count (ANC) ≥ 1500 cells/mm^3^
     - Platelet count ≥ 100,000 cells/mm^3^
     - Hemoglobin ≥ 9 g/dL or ≥ 5.6 mmol/L
     - Estimated glomerular filtration rate (GFR) ≥ 30 mL/min/1.73 m^2^ by Chronic Kidney Disease Epidemiology Collaboration equation
     - Serum total bilirubin ≤ 1.5 x upper limit of normal (ULN) (or < 3 x ULN in patients with Gilbert’s syndrome)
     - Prothrombin time/international normalized ratio (PT/INR) ≤ 1.5 x ULN unless the patient is receiving anti-coagulant therapy
     - Aspartate transaminase (AST) and alanine aminotransferase (ALT) ≤ 2.5 x ULN (or ≤ 5.0 x ULN in patients with liver metastases)
3. Hepatitis B virus (HBV) or hepatitis C virus (HCV) infection and meets the following criteria as applicable to the infection type:

For patients with inactive/asymptomatic carrier, chronic, or active HBV:
HBV DNA < 500 IU/mL (or 2500 copies/mL) at screening
NOTE: Patients with detectable hepatitis B surface antigen (HBsAg) or detectable HBV DNA should be managed per treatment guidelines. Patients receiving antivirals at screening should have been treated for > 2 weeks and have HBV < 500 IU/mL prior to randomization, and should continue treatment for at least 6 months after study drug treatment discontinuation

For patients with HCV:
Patients with detectable HCV RNA and who are receiving treatment at screening should remain on continuous, effective antiviral therapy during the study

1. Females of childbearing potential must have a negative serum pregnancy test within 7 days of randomization and must be willing to have additional pregnancy tests during the study. Females of childbearing potential must be willing to use highly effective methods of birth control for the duration of the study, and for at least 120 days after the last dose of tislelizumab and 180 days after the last dose of investigator-chosen chemotherapy (ICC)
2. Non-sterile males who have female sexual partner(s) of childbearing potential must use highly effective form of birth control for the duration of the study, and for at least 120 days after the last dose of tislelizumab and 180 days after the last dose of ICC
   - - A sterile male is defined as one for whom known azoospermia, in a semen sample examination, has been previously demonstrated as definitive evidence of infertility
     - Males with known ‘low sperm counts’ (consistent with ‘sub-fertility’) are not to be considered sterile for purposes of this study

Exclusion criteria

To be eligible to participate in this study, a patient was not permitted to meet any of the following exclusion criteria:

1. Ineligible for treatment with any of the treatments of protocol-specified chemotherapy
2. Receipt of two or more prior lines of systemic treatments for advanced/metastatic unresectable ESCC
3. Palliative radiation treatment for ESCC within 14 days of study treatment initiation (Cycle 1 Day 1 [C1D1])
4. History of gastrointestinal perforation and/or fistula or aorto-esophageal fistula within 6 months prior to randomization
5. Tumor invasion into organs located adjacent to the esophageal disease site (e.g., aorta or respiratory tract) at an increased risk of fistula in the study treatment assessed by investigator
6. Uncontrollable pleural effusion, pericardial effusion, or ascites requiring frequent drainage (recurrence within 2 weeks of intervention)
7. Current or past history of severe hypersensitivity reactions to other humanized monoclonal antibodies
8. Received prior therapies targeting programmed cell death protein 1 (PD-1) or programmed death-ligand 1 (PD-L1)
9. Has toxicities (as a result of prior anticancer therapy) which have not recovered to baseline or stabilized, except for adverse events (AEs) not considered a likely safety risk (e.g., alopecia, neuropathy, and specific laboratory abnormalities) exceptions are to be determined by the investigator in consultation with the medical monitor
10. Prior malignancy active within the previous 2 years before randomization (exceptions include the tumor under investigation in this study and surgically excised non-melanoma skin cancer, curatively treated carcinoma in situ of the cervix, localized prostate cancer treated with curative intent, curatively treated low-stage bladder cancer, ductal carcinoma in situ treated surgically with curative intent, or a malignancy diagnosed > 2 years ago, with no current evidence of disease and no therapy ≤ 2 years prior to randomization)
11. Active brain or leptomeningeal metastasis. Patients with equivocal findings or with confirmed brain metastases are eligible for enrollment provided that they are asymptomatic and radiologically stable without the need for corticosteroid treatment for at least 4 weeks prior to randomization. Computerized tomography/magnetic resonance imaging of the head at baseline is required for patients who are suspected to have central nervous system metastases
12. Has active autoimmune disease or history of autoimmune diseases at high risk for relapse

NOTE: Patients with following diseases may be enrolled if they meet all other eligibility criteria: controlled type I diabetes, hypothyroidism managed with hormone replacement therapy only, controlled celiac disease, skin diseases not requiring systemic treatment (such as vitiligo, psoriasis or alopecia), or diseases not expected to recur in the absence of external triggering factors

1. Has a condition requiring systemic treatment with either corticosteroids (> 10 mg daily prednisone or equivalents) or other immunosuppressive medications within 14 days prior to randomization
   - - Patients who have a history of organ transplant, including stem cell allograft, are not permitted to enroll
     - Adrenal replacement steroid dose ≤ 10 mg daily prednisone equivalents are permitted in the absence of active autoimmune disease
     - Patients are permitted to use topical, ocular, intra-articular, intranasal and inhalational corticosteroids (with minimal systemic absorption)
     - A brief course of corticosteroids for prophylaxis (e.g., contrast dye allergy) or for treatment of non-autoimmune conditions (e.g., delayed-type hypersensitivity reaction caused by contact allergen) is permitted
2. Undergone surgery requiring general anesthesia or epidural anesthesia within 28 days prior to randomization
3. Undergone surgery involving local anesthesia within 14 days prior to randomization. Exceptions to this exclusion criteria include:
   1. Placement of a central venous access device that requires up to a 3-day surveillance period
   2. A biopsy procedure performed under local anesthesia during the screening period that require a 7-day post procedure surveillance period
4. Received any radiopharmaceuticals (except for examination or diagnostic use of radiopharmaceuticals) within 42 days prior to randomization
5. Has received:
   1. Within 28 days or 5 half-lives (whichever is shorter but at least 14 days) of the first study drug administration: any chemotherapy, any immunotherapy (e.g., interleukin, interferon or thymosin) or any investigational therapies
   2. Within 14 days of the first study drug administration: any Chinese herbal medicine or Chinese patent medicines used to control cancer or boost immunity
6. Any serious or unstable pre-existing medical conditions (aside from malignancy exceptions specified above), psychiatric disorders, or other conditions that could interfere with the subject’s safety, obtaining informed consent, or compliance with study procedures
7. Receipt of a live vaccine within 4 weeks prior to C1D1

NOTE: Seasonal vaccines for influenza are generally inactivated vaccines and are allowed. Intranasal vaccines are live viruses and are not allowed

1. Known history of, or any evidence of interstitial lung disease, non-infectious pneumonitis, pulmonary fibrosis diagnosed based on imaging or clinical findings, or uncontrolled systemic diseases, including diabetes, hypertension, acute lung diseases, etc

NOTE: Patients with radiation pneumonitis may be randomized if the radiation pneumonitis has been confirmed as stable (beyond acute phase) and is unlikely to recur. Patients with severe but stable radiation-induced pneumonitis may be required to undergo routine pulmonary function studies

1. Has severe chronic or active infection (including tuberculosis infection, etc) requiring systemic antibacterial, antifungal or antiviral therapy, within 14 days prior to C1D1

NOTE: Patients who require systemic antiviral therapy for HBV are excepted

1. Known history of Human Immunodeficiency Virus (HIV)
2. Has any of the following cardiovascular risk factors:
   - - Ongoing cardiac chest pain, defined as moderate pain that limits instrumental activities of daily living
     - Symptomatic pulmonary embolism within 28 days before randomization
     - Any history of acute myocardial infarction within 6 months before randomization
     - Any history of heart failure meeting New York Heart Association Classification III or IV within 6 months before randomization
     - Any event of ventricular arrhythmia > grade 2 in severity within 6 months before randomization
     - Any history of cerebrovascular accident or transient ischemic attack within 6 months before randomization
     - Uncontrolled hypertension: systolic pressure ≥ 160 mmHg or diastolic pressure ≥ 100 mmHg despite anti-hypertension medications ≤ 28 days before randomization or first dose of study drug
     - Any episode of syncope or seizure ≤ 28 days before randomization

24. Severe malnutrition despite enteral or parenteral nutritional supplementation

25. Known, active alcohol or drug abuse or dependence

26. Pregnant or breastfeeding woman

## Study endpoints

### Primary endpoints

- - Overall survival (OS) in the intent-to-treat (ITT) analysis set – defined as the time from the date of randomization to the date of death due to any cause for all ITT patients

### Secondary endpoints

Key secondary endpoint

- - OS in the PD-L1-positive analysis set – defined as the time from the date of randomization until the date of death due to any cause for all PD-L1-positive patients

Other secondary endpoints

- - Overall response rate (ORR) – defined as the proportion of patients who had complete response (CR) or partial response (PR) assessed by the investigators per RECIST version 1.1
  - Progression-free survival (PFS) – defined as the time from the date of randomization to the date of first documentation of disease progression assessed by the investigators per RECIST version 1.1 or death, whichever occurs first
  - Duration of response (DoR) – measured from the time measurement criteria are first met for CR/PR (whichever is first recorded) until the first documentation of progression assessed by the investigators per RECIST v1.1 or death, whichever comes first
  - Health Related Quality of Life (HRQoL) assessment of the subject’s overall health status using European Organisation for Research and Treatment of Cancer (EORTC) Quality of Life Questionnaire (QLQ)-C30 index, global health status (GHS), and the functional and symptoms scales, the EORTC QLQ esophageal cancer module OES18 index score and the symptoms scale scores, and the generic health state instrument Euroqol 5D (EQ-5D-5L)
  - The incidence and severity of adverse events according to National Cancer Institute Common Terminology Criteria for Adverse Events version 4.03

### Exploratory endpoints

- - Disease control rate (DCR) – defined as the proportion of patients who have CR, PR and stable disease (SD) assessed by the Investigators per RECIST version 1.1
  - Pharmacokinetic endpoints: summary of serum concentration of tislelizumab to include, but not limited to, trough serum concentration (C_trough_)
  - Assessments of immunogenicity of tislelizumab to determine the incidence of anti-drug antibodies (ADA)
  - Predictive biomarkers (including but not limited to PD-L1 expression, gene expression profiling, tumor mutation burden, microsatellite instability [MSI] and tumor-infiltrated immune cells) and resistance mechanism

## Supplementary tables

**Table S1** Patient demographics and baseline characteristics in the Japanese subgroup and overall study patient population

|  | Overall study patient population^a^ | | Japanese patient subgroup | |
| --- | --- | --- | --- | --- |
|  | Tislelizumab  (*N*=256) | Chemotherapy  (*N*=256) | Tislelizumab  (*n*=25) | Chemotherapy  (*n*=25) |
| Age |  |  |  |  |
| Median, years (range) | 62.0 (40–86) | 63.0 (35–81) | 67.0 (47–83) | 63.0 (52–77) |
| < 65 years, n (%) | 157 (61.3) | 161 (62.9) | 8 (32.0) | 16 (64.0) |
| ≥ 65 years, n (%) | 99 (38.7) | 95 (37.1) | 17 (68.0) | 9 (36.0) |
| Sex, n (%) |  |  |  |  |
| Male | 217 (84.8) | 215 (84.0) | 20 (80.0) | 19 (76.0) |
| Female | 39 (15.2) | 41 (16.0) | 5 (20.0) | 6 (24.0) |
| Race, n (%) |  |  |  |  |
| Asian | 201 (78.5) | 207 (80.9) | 25 (100.0) | 25 (100.0) |
| Japanese | 25 (9.8) | 25 (9.8) | 25 (100.0) | 25 (100.0) |
| White/Caucasian | 53 (20.7) | 44 (17.2) | 0 (0.0) | 0 (0.0) |
| Black/African American | 0 (0.0) | 2 (0.8) | 0 (0.0) | 0 (0.0) |
| Other^b^ | 2 (0.8) | 3 (1.2) | 0 (0.0) | 0 (0.0) |
| Ethnicity |  |  |  |  |
| Hispanic or Latino | 2 (0.8) | 2 (0.8) | 0 (0.0) | 0 (0.0) |
| Not Hispanic or Latino | 252 (98.4) | 252 (98.4) | 25 (100.0) | 25 (100.0) |
| Unknown/not reported | 2 (0.8) | 2 (0.8) | 0 (0.0) | 0 (0.0) |
| ECOG performance status, n (%) |  |  |  |  |
| 0 | 66 (25.8) | 60 (23.4) | 14 (56.0) | 14 (56.0) |
| 1 | 190 (74.2) | 196 (76.6) | 11 (44.0) | 11 (44.0) |
| PD-L1 score^c^, n (%) |  |  |  |  |
| ≥ 10% | 80 (31.3) | 62 (24.2) | 10 (40.0) | 6 (24.0) |
| < 10% | 100 (39.1) | 122 (47.7) | 4 (16.0) | 6 (24.0) |
| Unknown/missing | 76 (29.7)^d^ | 72 (28.1)^d^ | 11 (44.0)^e^ | 13 (52.0)^e^ |
| Smoking status, n (%) |  |  |  |  |
| Never | 68 (26.6) | 63 (24.6) | 3 (12.0) | 2 (8.0) |
| Former/current | 188 (73.5) | 192 (75.0) | 22 (88.0) | 23 (92.0) |
| Missing | 0 (0.0) | 1 (0.4) | 0 (0.0) | 0 (0.0) |
| Previous anti-cancer interventions/therapies, n (%) |  |  |  |  |
| Surgery | 94 (36.7) | 99 (38.7) | 11 (44.0) | 8 (32.0) |
| Radiotherapy | 169 (66.0) | 163 (63.7) | 20 (80.0) | 15 (60.0) |
| Platinum-based chemotherapy | 249 (97.3) | 252 (98.4) | 23 (92.0) | 25 (100.0) |
| Disease stage at study entry, n (%) |  |  |  |  |
| Locally advanced | 5 (2.0) | 20 (7.8) | 0 (0.0) | 4 (16.0) |
| Metastatic | 251 (98.0) | 236 (92.2) | 25 (100.0) | 21 (84.0) |

*ECOG* Eastern Cooperative Oncology Group, *ITT* intent-to-treat, *PD-L1* programmed death-ligand 1

^a^Most data for the overall population were previously reported by Shen et al. J Clin Oncol. 2022 [14]

^b^Including categories of ‘not reported’, ‘unknown’ and ‘other’

^c^Visually estimated combined positive score

^d^‘Unknown’ score referred to patients without sample collection or not evaluable at baseline

^e^‘Missing’ score refers to the patients without sample collection, not evaluable at baseline or scored with unqualified sample

Data are presented for the overall ITT patient population (which comprised all randomized patients, analyzed according to their randomized treatment arm), and the Japanese patient subgroup of the ITT population

**Table S2** Post-study treatment anti-cancer interventions/therapies in the Japanese patient subgroup

|  | Tislelizumab  (*n*=25) | Chemotherapy  (*n*=25) |
| --- | --- | --- |
| Patients with any subsequent anti-cancer interventions/therapies, n (%) | 17 (68.0) | 12 (48.0) |
| Procedure, radiotherapy or surgery | 4 (16.0) | 3 (12.0) |
| Systemic therapy | 15 (60.0) | 11 (44.0) |
| Immunotherapy | 4 (16.0) | 6 (24.0) |

Data are presented for the Japanese subgroup of the intent-to-treat population, which comprised all randomized patients, analyzed according to their randomized treatment arm

**Table S3** Summary of efficacy outcomes in the overall study patient population and Japanese patient subgroup

|  | Overall study patient population^a^ | | Japanese patient subgroup | |
| --- | --- | --- | --- | --- |
|  | Tislelizumab | Chemotherapy | Tislelizumab | Chemotherapy |
| **Overall survival** |  |  |  |  |
| *Intent-to-treat population* | *N=256* | *N=256* | *n=25* | *n=25* |
| Events, n (%) of patients | 197 (77.0) | 213 (83.2) | 19 (76.0) | 20 (80.0) |
| Median, months (95% CI) | 8.6 (7.5, 10.4) | 6.3 (5.3, 7.0) | 9.8 (7.5, 17.3) | 7.6 (4.1, 10.5) |
| Hazard ratio (95% CI)^b^ | 0.70 (0.57, 0.85) | | 0.59 (0.31, 1.12) | |
| Overall survival rate, % of patients |  |  |  |  |
| 6 months | 62.3 | 51.8 | 72.0 | 59.7 |
| 12 months | 37.4 | 23.7 | 46.6 | 18.4 |
| *PD-L1 score ≥ 10% population^c^* | *n = 80* | *n = 62* | *n = 10* | *n = 6* |
| Events, n (%) of patients | 54 (67.5) | 53 (85.5) | 7 (70.0) | 5 (83.0) |
| Median, months (95% CI) | 10.0 (8.5, 15.1) | 5.1 (3.8, 8.2) | 12.5 (4.3, NE) | 2.9 (2.3, NE) |
| Hazard ratio (95% CI)^b^ | 0.49 (0.33, 0.74) | | 0.31 (0.09, 1.03) | |
| Overall survival rate, % of patients |  |  |  |  |
| 6 months | 66.3 | 48.3 | 70.0 | 16.7 |
| 12 months | 42.5 | 24.2 | 50.0 | 16.7 |
| **Progression-free survival** |  |  |  |  |
| *Intent-to-treat population* | *N=256* | *N=256* | *n=25* | *n=25* |
| Events, n (%) of patients | 223 (87.1) | 180 (70.3) | 21 (84.0) | 21 (84.0) |
| Median, months (95% CI) | 1.6 (1.4, 2.7) | 2.1 (1.5, 2.7) | 3.6 (2.0, 7.4) | 1.7 (1.4, 2.8) |
| Hazard ratio (95% CI)^b^ | 0.83 (0.67, 1.01) | | 0.50 (0.27, 0.95) | |
| Progression-free survival rate, % of patients |  |  |  |  |
| 6 months | 21.7 | 14.9 | 32.0 | 13.3 |
| 12 months | 12.7 | 1.9 | 20.0 | 0.0 |
| **Tumor response** |  |  |  |  |
| *Intent-to-treat population* | *N=256* | *N=256* | *n=25* | *n=25* |
| Objective response rate, n (%) of patients | 52 (20.3) | 25 (9.8) | 8 (32.0) | 5 (20.0) |
| 95% CI | 15.6, 25.8 | 6.4, 14.1 | 14.9, 53.5 | 6.8, 40.7 |
| Odds ratio (95% CI)^d^ | 2.39 (1.42, 4.01) | | 1.88 (0.52, 6.84) | |
| Best overall response, n (%) of patients |  |  |  |  |
| Complete response | 5 (2.0) | 1 (0.4) | 1 (4.0) | 1 (4.0) |
| Partial response | 47 (18.4) | 24 (9.4) | 7 (28.0) | 4 (16.0) |
| Stable disease | 68 (26.6) | 82 (32.0) | 10 (40.0) | 6 (24.0) |
| Progressive disease | 116 (45.3) | 86 (33.6) | 7 (28.0) | 11 (44.0) |
| Not evaluable^e^ | 1 (0.4) | 3 (1.2) | 0 (0.0) | 0 (0.0) |
| Not assessable^f^ | 19 (7.4) | 60 (23.4) | 0 (0.0) | 3 (12.0) |
| Median duration of response, months (95% CI) | 7.1 (4.1, 11.3) | 4.0 (2.1, 8.2) | 8.8 (2.9, NE) | 2.6 (1.1, NE) |
| Patients with ongoing response, n/N (%) | 10/52 (19.2) | 0/25 (0.0) | 2/8 (25.0) | 0/5 (0.0) |

*CI* confidence interval, *ECOG* Eastern Cooperative Oncology Group, *NE* not estimable, , *PD-L1* programmed death-ligand 1

^a^Data for the overall population were previously reported by Shen et al. J Clin Oncol. 2022 [14]

^b^Hazard ratios were based on a stratified Cox regression model for the overall patient population (including treatment as a covariate and ECOG performance status and chemotherapy option as strata) and without considering stratification for the Japanese patient subgroup (including treatment as a covariate)

^c^Visually estimated combined positive score

^d^The odds ratio for objective response rate between arms was calculated using a stratified Cochran-Mantel-Haenszel method for the overall patient population (with ECOG performance status and chemotherapy option as strata) and without considering stratification for the Japanese patient population

^e^Not evaluable was based on Response Evaluation Criteria in Solid Tumors version 1.1

^f^Not assessable refers to patients with no post-baseline tumor assessment, including those who discontinued the study or died without having a post-baseline tumor assessment

The intent-to-treat population comprised all randomized patients, analyzed according to their randomized treatment. Medians were estimated by the Kaplan–Meier method, with 95% CIs estimated using the Brookmeyer and Crowley method. For objective response rates, the two-sided 95% CIs were calculated using the Clopper-Pearson method. Objective response and duration of response were based on unconfirmed responses. Survival rates were estimated by the Kaplan–Meier method

**Table S4** Treatment-related treatment-emergent adverse events leading to dose modification in the Japanese patient subgroup

| n (%) | Tislelizumab  (*n*=25) | Chemotherapy  (*n*=23) |
| --- | --- | --- |
| Any TRAE leading to dose modification^a^ | 4 (16.0) | 16 (69.6) |
| TRAEs leading to dose modification by system organ class and preferred term |  |  |
| Metabolism and nutrition disorders | 2 (8.0) | 1 (4.3) |
| Hyponatremia | 1 (4.0) | 0 (0.0) |
| Type 1 diabetes mellitus | 1 (4.0) | 0 (0.0) |
| Decreased appetite | 0 (0.0) | 1 (4.3) |
| Endocrine disorders | 1 (4.0) | 0 (0.0) |
| Hyperthyroidism | 1 (4.0) | 0 (0.0) |
| General disorders and administration site conditions | 1 (4.0) | 2 (8.7) |
| Fatigue | 1 (4.0) | 0 (0.0) |
| Malaise | 0 (0.0) | 2 (8.7) |
| Blood and lymphatic system disorders | 0 (0.0) | 2 (8.7) |
| Anemia | 0 (0.0) | 1 (4.3) |
| Febrile neutropenia | 0 (0.0) | 1 (4.3) |
| Gastrointestinal disorders | 0 (0.0) | 1 (4.3) |
| Stomatitis | 0 (0.0) | 1 (4.3) |
| Investigations | 0 (0.0) | 12 (52.2) |
| Neutrophil count decreased | 0 (0.0) | 11 (47.8) |
| White blood cell count decreased | 0 (0.0) | 3 (13.0) |
| Nervous system disorders | 0 (0.0 | 1 (4.3) |
| Peripheral sensory neuropathy | 0 (0.0 | 1 (4.3) |

*TRAE* treatment-related treatment-emergent adverse event

^a^Dose modification included dose held, dose interruption and dose reduction for the chemotherapy arm, and dose held and dose interruption for the tislelizumab arm. All data are presented as number of patients with at least one event (percentage of patients). Data are presented for the Japanese subgroup of the safety population, which comprised all randomized patients who received at least one dose of a study drug, analyzed according to the actual study drug received. Adverse events were coded using Medical Dictionary for Regulatory Activities version 23.0. TRAEs include events that were considered by the investigator to be related to study drug or with a missing causality

**Table S5** Infusion-related reactions in the Japanese patient subgroup

|  | Tislelizumab  (*n*=25) | | Chemotherapy  (*n*=23) | |
| --- | --- | --- | --- | --- |
| n (%) | All grades | ≥ Grade 3 | All grades | ≥ Grade 3 |
| At least one infusion-related reaction | 3 (12.0) | 0 (0.0) | 0 (0.0) | 0 (0.0) |
| Infusion-related reactions by system organ class and preferred term |  |  |  |  |
| General disorders and administration site conditions | 2 (8.0) | 0 (0.0) | 0 (0.0) | 0 (0.0) |
| Pyrexia | 2 (8.0) | 0 (0.0) | 0 (0.0) | 0 (0.0) |
| Skin and subcutaneous tissue disorders | 1 (4.0) | 0 (0.0) | 0 (0.0) | 0 (0.0) |
| Rash | 1 (4.0) | 0 (0.0) | 0 (0.0) | 0 (0.0) |

All data are presented as number of patients with at least one event (percentage of patients). Data are presented for the Japanese subgroup of the safety population, which comprised all randomized patients who received at least one dose of a study drug, analyzed according to the actual study drug received. Adverse event grades were based on National Cancer Institute Common Terminology Criteria for Adverse Events version 4.03. Adverse events were coded using Medical Dictionary for Regulatory Activities version 23.0

**Table S6** Summary of treatment-emergent adverse event incidence in the overall study patient population and Japanese patient subgroup

|  | Overall study patient population^a^ | | Japanese patient subgroup | |
| --- | --- | --- | --- | --- |
| n (%) | Tislelizumab  (*N*=255) | Chemotherapy  (*N*=240) | Tislelizumab  (*n*=25) | Chemotherapy  (*n*=23) |
| Any TEAE | 244 (95.7) | 236 (98.3) | 24 (96.0) | 22 (95.7) |
| ≥ Grade 3 TEAE | 118 (46.3) | 163 (67.9) | 11 (44.0) | 16 (69.6) |
| Serious TEAE | 105 (41.2) | 105 (43.8) | 9 (36.0) | 10 (43.5) |
| TEAE leading to treatment discontinuation | 49 (19.2) | 64 (26.7) | 2 (8.0) | 4 (17.4) |
| TEAE leading to dose modification^b^ | 58 (22.7) | 115 (47.9) | 8 (32.0) | 16 (69.6) |
| TEAE leading to death^c^ | 14 (5.5) | 14 (5.8) | 1 (4.0) | 1 (4.3) |
| Any TRAE | 187 (73.3) | 225 (93.8) | 17 (68.0) | 22 (95.7) |
| ≥ Grade 3 TEAE | 48 (18.8) | 134 (55.8) | 6 (24.0) | 11 (47.8) |
| Serious TEAE | 36 (14.1) | 47 (19.6) | 4 (16.0) | 2 (8.7) |
| TEAE leading to treatment discontinuation | 17 (6.7) | 33 (13.8) | 2 (8.0) | 2 (8.7) |
| TEAE leading to dose modification^b^ | 34 (13.3) | 106 (44.2) | 4 (16.0) | 16 (69.6) |
| TEAE leading to death^c^ | 7 (2.7) | 8 (3.3) | 0 (0.0) | 0 (0.0) |
| Incidence of most common TRAEs occurring in ≥ 10% of patients per treatment arm by preferred term^d^ |  |  |  |  |
| Aspartate aminotransferase increased | 29 (11.4) | 9 (3.8) | 2 (8.0) | 0 (0.0) |
| Anemia | 28 (11.0) | 83 (34.6) | 0 (0.0) | 2 (8.7) |
| Hypothyroidism | 26 (10.2) | 0 (0.0) | 3 (12.0) | 0 (0.0) |
| Fatigue | 19 (7.5) | 33 (13.8) | 3 (12.0) | 5 (21.7) |
| Decreased appetite | 16 (6.3) | 75 (31.3) | 0 (0.0) | 5 (21.7) |
| Pruritus | 15 (5.9) | 8 (3.3) | 3 (12.0) | 1 (4.3) |
| Diarrhea | 14 (5.5) | 66 (27.5) | 1 (4.0) | 1 (4.3) |
| Pneumonitis | 12 (4.7) | 3 (1.3) | 3 (12.0) | 2 (8.7) |
| Asthenia | 12 (4.7) | 28 (11.7) | 0 (0.0) | 0 (0.0) |
| Malaise | 10 (3.9) | 35 (14.6) | 3 (12.0) | 4 (17.4) |
| Weight decreased | 8 (3.1) | 25 (10.4) | 0 (0.0) | 2 (8.7) |
| Arthralgia | 7 (2.7) | 11 (4.6) | 2 (8.0) | 6 (26.1) |
| Nausea | 7 (2.7) | 66 (27.5) | 0 (0.0) | 0 (0.0) |
| Leukopenia | 7 (2.7) | 30 (12.5) | 0 (0.0) | 0 (0.0) |
| White blood cell count decreased | 5 (2.0) | 98 (40.8) | 1 (4.0) | 12 (52.2) |
| Stomatitis | 4 (1.6) | 14 (5.8) | 1 (4.0) | 6 (26.1) |
| Vomiting | 4 (1.6) | 43 (17.9) | 0 (0.0) | 0 (0.0) |
| Constipation | 4 (1.6) | 25 (10.4) | 0 (0.0) | 1 (4.3) |
| Neutrophil count decreased | 3 (1.2) | 94 (39.2) | 0 (0.0) | 13 (56.5) |
| Neutropenia | 2 (0.8) | 31 (12.9) | 0 (0.0) | 2 (8.7) |
| Peripheral sensory neuropathy | 2 (0.8) | 22 (9.2) | 0 (0.0) | 7 (30.4) |
| Myalgia | 2 (0.8) | 14 (5.8) | 0 (0.0) | 3 (13.0) |
| Alopecia | 0 (0.0) | 42 (17.5) | 0 (0.0) | 11 (47.8) |

*TEAE* treatment-emergent adverse event, *TRAE* treatment-related treatment-emergent adverse event

^a^Most data for the overall population were previously reported by Shen et al. J Clin Oncol. [14]

^b^Dose modification included dose held, dose interruption and dose reduction for the chemotherapy arm, and dose held and dose interruption for the tislelizumab arm

^c^Deaths caused by disease progression were excluded

^d^Data presented are TEAEs reported in ≥ 10% of patients per treatment arm in either the overall study patient population or the Japanese patient subgroup, ordered by decreasing incidence in the tislelizumab arm of the overall study patient population

All data are presented as number of patients with at least one event (percentage of patients). Data are presented for the overall safety population (which comprised all randomized patients who received at least one dose of a study drug, analyzed according to the actual study drug received), and the Japanese patient subgroup of the safety population. TRAEs include TEAEs that were considered by the investigator to be related to study drug or TEAEs with a missing causality. Adverse event grades were based on National Cancer Institute Common Terminology Criteria for Adverse Events version 4.03. Adverse events were coded using Medical Dictionary for Regulatory Activities version 23.0

**Table S7** Tislelizumab serum concentration in the tislelizumab arm in the overall study patient population and Japanese patient subgroup

|  | Overall study patient population | Japanese patient subgroup |
| --- | --- | --- |
| Tislelizumab serum concentration, μg/mL |  |  |
| C1D1: post-dose |  |  |
| N | 245 | 25 |
| Mean concentration (CV%) | 66.1 (21.0) | 64.8 (23.2) |
| SD | 13.9 | 15.0 |
| Geometric mean (CV%) | 64.6 (21.5) | 63.2 (23.9) |
| Median (range) | 64.8 (22.3–129.0) | 62.7 (31.9–108.0) |
| C2D1: pre-dose |  |  |
| N | 212 | 24 |
| Mean concentration (CV%) | 17.3 (27.9) | 18.2 (24.5) |
| SD | 4.8 | 4.4 |
| Geometric mean (CV%) | 16.6 (29.1) | 17.6 (26.0) |
| Median (range) | 16.9 (5.7–36.7) | 18.3 (10.1–27.5) |
| C5D1: pre-dose |  |  |
| N | 94 | 13 |
| Mean concentration (CV%) | 40.6 (33.2) | 45.6 (31.4) |
| SD | 13.4 | 14.3 |
| Geometric mean (CV%) | 38.5 (33.9) | 43.7 (30.6) |
| Median (range) | 38.4 (18.5–80.5) | 42.9 (27.3–78.9) |
| C5D1: post-dose |  |  |
| N | 93 | 13 |
| Mean concentration (CV%) | 109.4 (21.4) | 117.7 (23.1) |
| SD | 23.4 | 27.2 |
| Geometric mean (CV%) | 107.0 (21.5) | 115.1 (21.4) |
| Median (range) | 107.0 (62.8–184.0) | 112.0 (86.0–184.0) |
| C9D1: pre-dose |  |  |
| N | 53 | 9 |
| Mean concentration (CV%) | 48.7 (35.6) | 60.1 (33.6) |
| SD | 17.3 | 20.2 |
| Geometric mean (CV%) | 45.4 (40.9) | 57.3 (33.2) |
| Median (range) | 46.4 (11.7–93.1) | 54.0 (36.5–93.1) |
| C17D1: pre-dose |  |  |
| N | 24 | 7 |
| Mean concentration (CV%) | 50.1 (42.7) | 46.2 (67.4) |
| SD | 21.4 | 31.1 |
| Geometric mean (CV%) | 44.1 (64.4) | 33.9 (121.5) |
| Median (range) | 48.5 (7.1–83.4) | 58.0 (7.1–83.4) |

*C* cycle, *CV* coefficient of variation, *D* Day, *SD* standard deviation

Data are presented for the overall pharmacokinetic analysis set (which comprised all patients who received at least one dose of tislelizumab per the protocol, for whom any post-dose pharmacokinetic data were available), and the Japanese patient subgroup of the pharmacokinetic analysis set. Post-dose assessments were within 30 minutes after the end of infusion

Tislelizumab serum concentrations for C1D1 predose were all below limit of quantification for the overall study population, including the Japanese patient subgroup

## Supplementary Figures

**Fig. S1** Patient flow in the Japanese patient subgroup

**
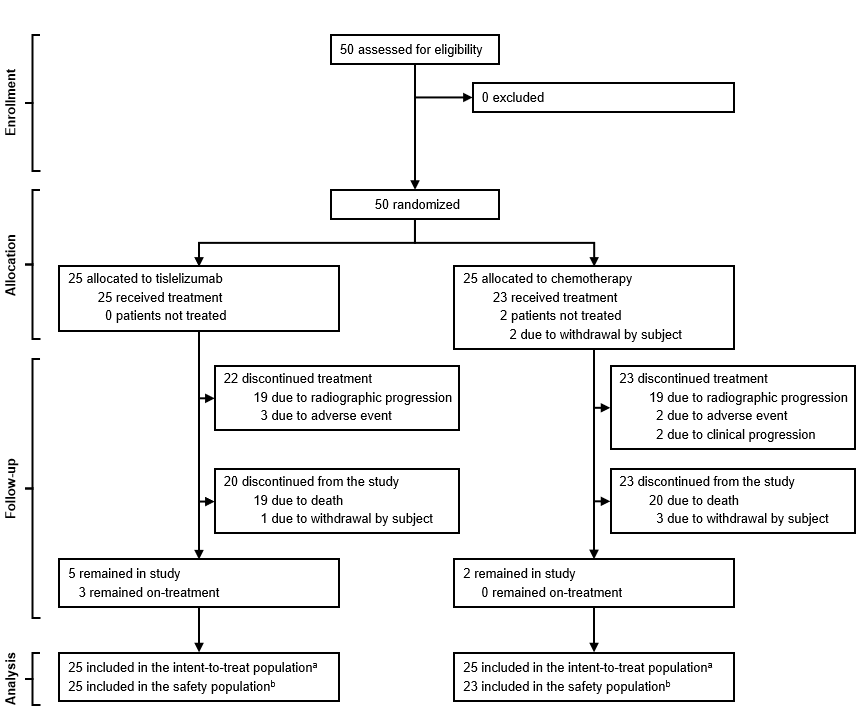
**

^a^The intent-to-treat population comprised all randomized patients, analyzed according to their randomized treatment. ^b^The safety population comprised all randomized patients who received at least one dose of a study drug, analyzed according to the actual study drug received

**Fig. S2** Overall survival in the PD-L1-positive population for the Japanese patient subgroup


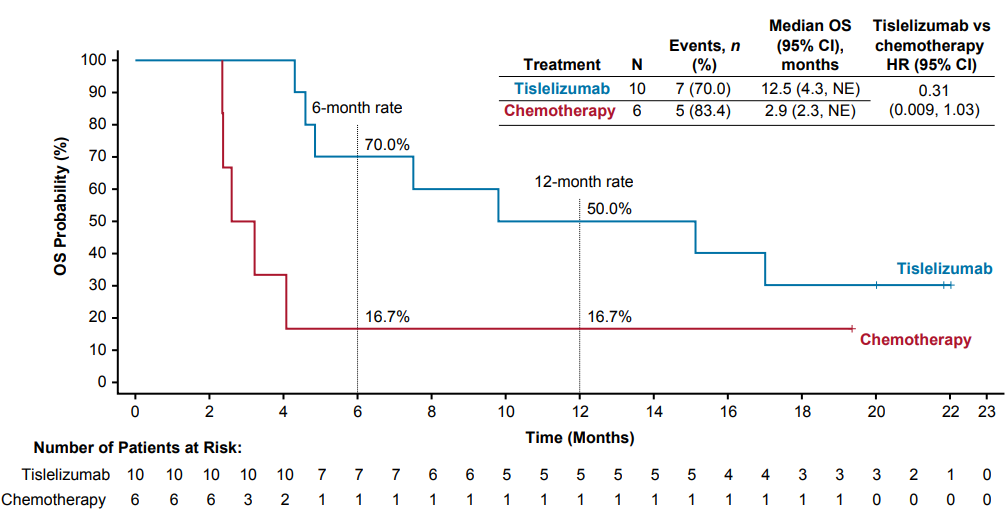


*CI* confidence interval, *HR* hazard ratio, *NE* not estimable, *OS* overall survival

Data are presented for the PD-L1-positive population within the Japanese patient subgroup. The PD-L1-positive population comprised patients with PD-L1 score ≥ 10% using the VENTANA SP263 assay. The graph presents the Kaplan–Meier survival plot, with OS rates (cumulative probability of OS) at selected timepoints estimated by Kaplan–Meier method. The tabular data present: hazard ratios based on an unstratified Cox regression model including treatment as a covariate; medians estimated by the Kaplan–Meier method with 95% CIs estimated using the Brookmeyer and Crowley method

**Fig. S3** Observed scores and change from baseline in QLQ-C30 scores (**a**), QLQ-OES18 scores (**b**) and EQ-5D-5L scores (**c**) at Cycle 4 (or Week 12) in the overall study patient population and Japanese patient subgroup

**(a)** **
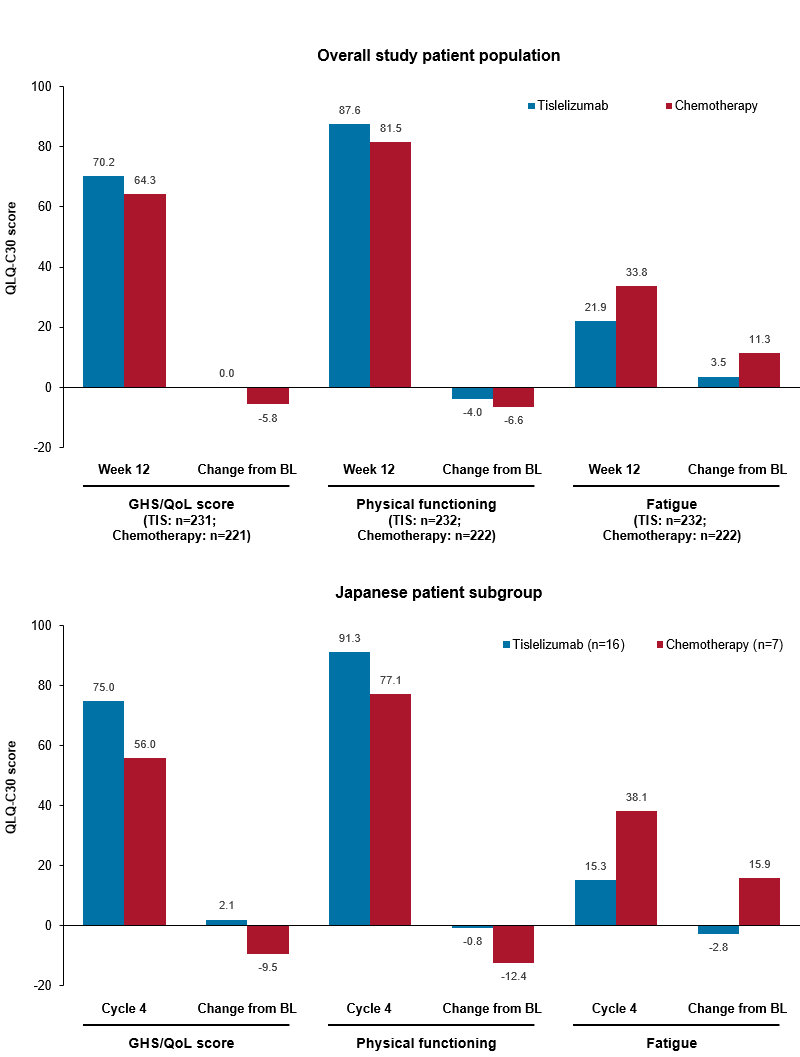
**

**(b)** **
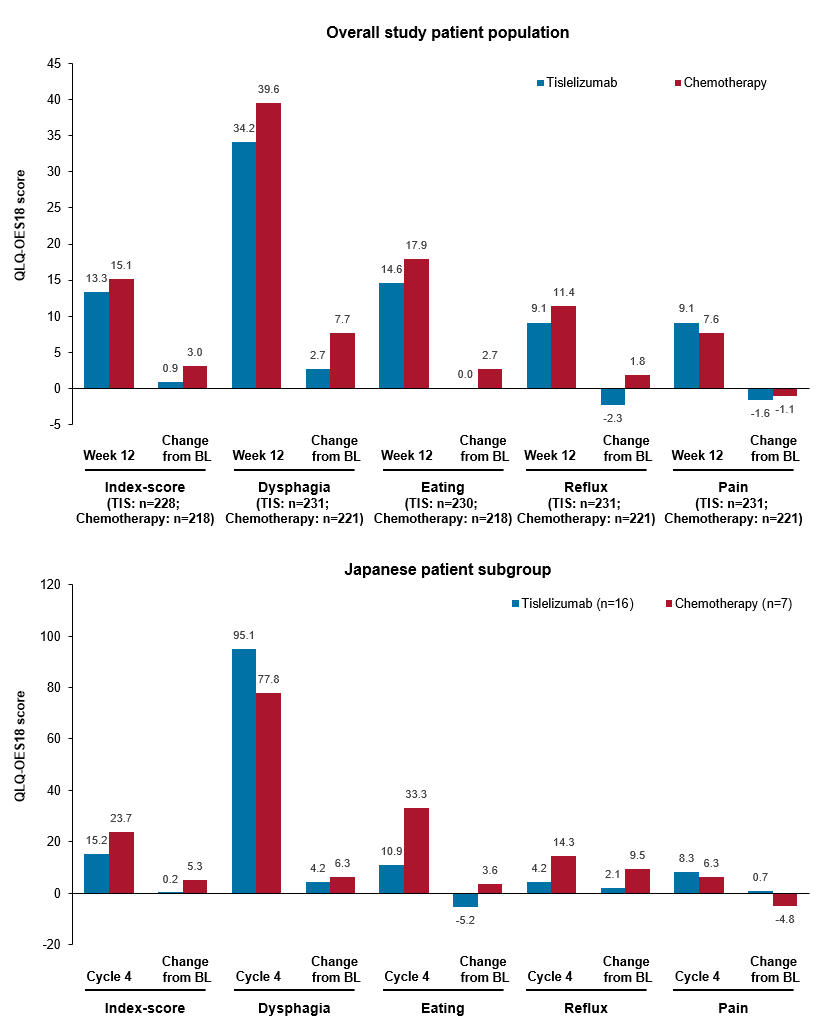
**

**(c)** **
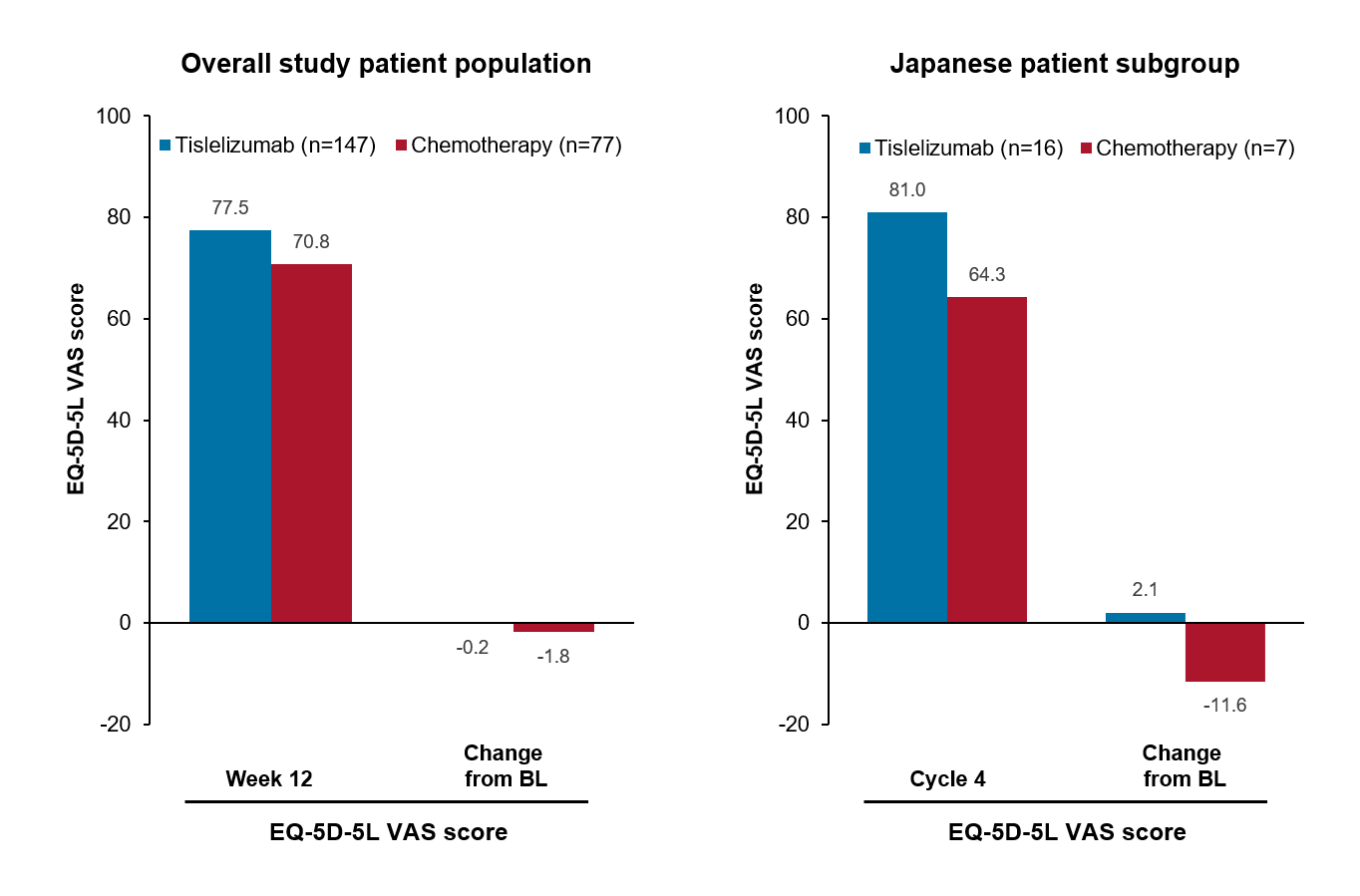
**

*BL* baseline, *EQ-5D-5L* EuroQoL-5-dimensions-5 levels, *GHS* global health score, *ITT* intent-to-treat, *LS* least squares, *QLQ-C30* Core Quality of Life Questionnaire Core 30 items, *QLQ-OES18* Quality of Life Questionnaire Oesophageal Cancer Module 18 items, *QoL* quality of life, *VAS* visual analogue scale

Data for the overall population were previously reported by Van Cutsem et al. ESMO Open. 2022;7(4):100517 [14]

Data are presented for the overall study ITT population (which included all randomized patients, analyzed according to their randomized treatment arm) and the Japanese patient subgroup of the ITT population. For the QLQ-C30 and QLQ-OES18 assessments, higher scores on the GHS/QoL scale and physical function scale indicate a better outcome, while lower symptom scores (fatigue, dysphagia, eating, reflux, and pain) indicate better outcomes
